# *N*′-[(*E*)-Furan-2-yl­methyl­idene]pyridine-3-carbohydrazide

**DOI:** 10.1107/S1600536811046381

**Published:** 2011-11-12

**Authors:** Jessy Emmanuel, M. Sithambaresan, M. R. Prathapachandra Kurup

**Affiliations:** a Department of Applied Chemistry, Cochin University of Science and Technology, Kochi 682 022, India; b Department of Chemistry, Faculty of Science, Eastern University, Sri Lanka, Chenkalady, Sri Lanka

## Abstract

The title compound, C_11_H_9_N_3_O_2_, exists in the *E* conformation with respect to the azomethane C=N bond, and has the keto form. There are two independent mol­ecules in the asymmetric unit and each of these features a slight slanting of the pyridine and furan rings, which form a dihedral angle of 14.96 (10)° in one of the mol­ecules and 5.53 (10)° in the other. The crystal structure is stabilized by N—H⋯O and N—H⋯N hydrogen bonds, weak C—H⋯O and C—H⋯N hydrogen bonds and C—H⋯π inter­actions and π–π inter­actions [shortest centroid–centroid distance = 3.7864 (15) Å].

## Related literature

For applications of carbohydrazide in non-linear optics and mol­ecular sensing, see: Bakir & Brown (2002 ▶). For the synthesis of related compounds, see: Fun *et al.* (2008 ▶); Neema & Kurup (2011 ▶). For similar structures, see: Nancy *et al.* (2011 ▶). For standard bond-length data, see: Allen *et al.* (1987 ▶).
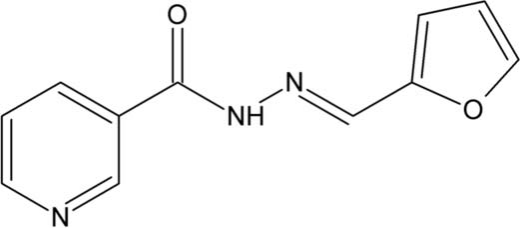


## Experimental

### 

#### Crystal data

C_11_H_9_N_3_O_2_
*M* *_r_* = 215.21
Triclinic, 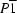

*a* = 9.441 (2) Å
*b* = 10.237 (3) Å
*c* = 11.023 (2) Å
α = 75.10 (2)°
β = 85.413 (19)°
γ = 84.11 (2)°
*V* = 1022.5 (4) Å^3^
*Z* = 4
Mo *K*α radiation
μ = 0.10 mm^−1^
*T* = 150 K
0.26 × 0.21 × 0.18 mm

#### Data collection

Bruker P4 diffractometer
Absorption correction: multi-scan (*CrysAlis RED*; Oxford Diffraction, 2006 ▶) *T*_min_ = 0.975, *T*_max_ = 0.982
9430 measured reflections
3589 independent reflections
2702 reflections with *I* > 2σ(*I*)
*R*_int_ = 0.023

#### Refinement

*R*[*F* ^2^ > 2σ(*F* ^2^)] = 0.040
*wR*(*F* ^2^) = 0.117
*S* = 1.09
3589 reflections
298 parameters
2 restraints
H atoms treated by a mixture of independent and constrained refinement
Δρ_max_ = 0.22 e Å^−3^
Δρ_min_ = −0.29 e Å^−3^

### 

Data collection: *CrysAlis CCD* (Oxford Diffraction, 2006 ▶); cell refinement: *CrysAlis RED* (Oxford Diffraction, 2006 ▶); data reduction: *CrysAlis RED*; program(s) used to solve structure: *SHELXS97* (Sheldrick, 2008 ▶); program(s) used to refine structure: *SHELXL97* (Sheldrick, 2008 ▶); molecular graphics: *SHELXTL* (Sheldrick, 2008 ▶) and *ORTEP-3* (Farrugia, 1997 ▶); software used to prepare material for publication: *SHELXL97* and *publCIF* (Westrip, 2010 ▶).

## Supplementary Material

Crystal structure: contains datablock(s) global, I. DOI: 10.1107/S1600536811046381/tk5012sup1.cif

Structure factors: contains datablock(s) I. DOI: 10.1107/S1600536811046381/tk5012Isup2.hkl

Supplementary material file. DOI: 10.1107/S1600536811046381/tk5012Isup3.cml

Additional supplementary materials:  crystallographic information; 3D view; checkCIF report

## Figures and Tables

**Table 1:** Hydrogen-bond geometry (Å, °) *Cg*1 and *Cg*2 are the centroids of the O4/C19–C22 and O2/C8–C11 rings, respectively.

*D*—H⋯*A*	*D*—H	H⋯*A*	*D*⋯*A*	*D*—H⋯*A*
N5—H5*N*⋯N1^i^	0.86 (2)	2.10 (2)	2.944 (2)	169 (2)
N2—H2*N*⋯O3	0.89 (1)	2.08 (2)	2.9017 (19)	154 (2)
C21—H21⋯N3^ii^	0.93	2.58	3.410 (3)	149
C16—H16⋯N1^i^	0.93	2.53	3.370 (3)	150
C11—H11⋯O1^iii^	0.93	2.51	3.365 (2)	153
C2—H2⋯O3^iv^	0.93	2.49	3.116 (2)	125
C10—H10⋯*Cg*1^v^	0.93	2.78	3.594 (2)	146
C12—H12⋯*Cg*2^vi^	0.93	2.75	3.520 (2)	141

Symmetry codes: (i) 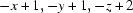; (ii) 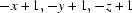; (iii) 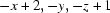; (iv) 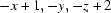; (v) 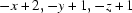; (vi) 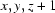.

**Fig. 1.**
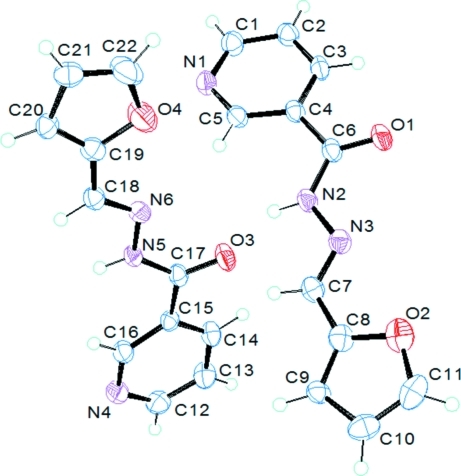
The molecular structure of (I) showing the atom-labelling scheme and displacement ellipsoids at the 50% probability level.

**Fig. 2.**
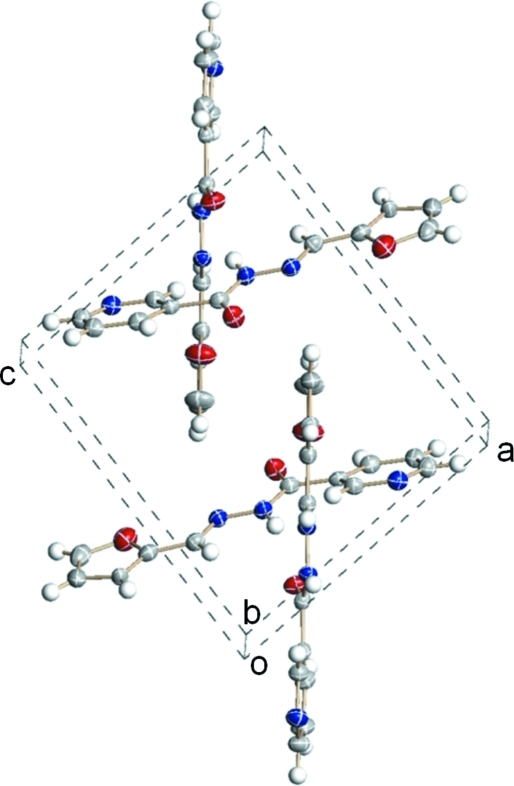
A view of the unit cell for (I).

## supplementary crystallographic information

### Comment

Derivatives of pyridine-3-carbohydrazide and their metal complexes possess pronounced biological activities. They also contain versatile binding properties and the presence of the carbonyl-O atom promotes the the formation of a chelate binding center (Neema & Kurup, 2011). Derivatives of pyridine-3-carbohydrazide and their metal complexes have received considerable attention during the last decade because of their versatile applications in nonlinear optics and molecular sensing (Bakir & Brown, 2002). The present report is an extension of earlier studies in this area (Nancy *et al.*, 2011).

The title compound, (I), crystallizes in triclinic space group P1.There are two independent molecules (Fig. 1) in the asymmetric unit with almost the same bond length and bond angle, and therefore the detailed description can be limited to one of these molecules. The molecule adopts an *E* configuration with respect to C7═N3 bond and it exists in the keto form with C6═O1 bond length of 1.226 (2) Å which is very close to a formal C═O bond length [1.21 Å] (Allen *et al.*, 1987). The dihedral angle between pyridine and furan rings is 14.96 (10)°. The O1 and N3 atoms are *syn* with respect to the C6—N2 bond having a torsion angle of -2.0 (3)°. The molecule is almost planar with maximum deviation of 0.265 (2) Å for atom C2; the other molecule has the maximum deviation of 0.212 (1) Å for the atom O3 from its least-squares plane.

Conventional N—H···O,N hydrogen bonds are present, Table 1, Moreover, there are non-conventional intermolecular interactions present in the crystal structure, Table 1, which contribute to the formation of a 3-D network.

Fig. 2 shows a partial packing diagram. The molecules shown participate in C–H···π interactions formed between the H atoms attached at the C10 and C12 atoms and the furan rings, Table 1. The presence of π–π interactions, with centroid-centroid distances of 3.7864 (15), 3.7864 (15), 3.7274 (15) and 3.7273 (15) Å between the rings, is also noted.

### Experimental

The title compound, (I), was prepared by adapting a reported procedure (Fun *et al.*, 2008) by refluxing a mixture of a methanol solutions of furan-2-carboxaldehyde (0.960 g, 10 mmol) and pyridine-3-carbohydrazide (1.370 g, 10 mmol) for 3 h. The formed crystals were collected and recrystallized from a mixture of ethanol and dimethylformamide (1:1 *v*/*v*). Light-green crystals were obtained.

### Refinement

All C-bound H atoms were placed in calculated positions with C—H = 0.93 Å, and with *U*_iso_=1.2*U*_eq_(C). The N-bound H atoms were refined with N—H = 0.88±0.1 Å and free *U*_iso_.

### Crystal data

**Table:**

C_11_H_9_N_3_O_2_	*Z* = 4
*M**_r_* = 215.21	*F*(000) = 448
Triclinic, *P*1	*D*_x_ = 1.398 Mg m^−^^3^
Hall symbol: -P 1	Mo *K*α radiation, λ = 0.71073 Å
*a* = 9.441 (2) Å	Cell parameters from 2702 reflections
*b* = 10.237 (3) Å	θ = 3.0–25.0°
*c* = 11.023 (2) Å	µ = 0.10 mm^−^^1^
α = 75.10 (2)°	*T* = 150 K
β = 85.413 (19)°	Block, light-green
γ = 84.11 (2)°	0.26 × 0.21 × 0.18 mm
*V* = 1022.5 (4) Å^3^	

### Data collection

**Table:**

Bruker P4 diffractometer	3589 independent reflections
Radiation source: Enhance (Mo) X-ray Source	2702 reflections with *I* > 2σ(*I*)
graphite	*R*_int_ = 0.023
Detector resolution: 8.33 pixels mm^-1^	θ_max_ = 25.0°, θ_min_ = 3.0°
ω scans	*h* = −11→11
Absorption correction: multi-scan (*CrysAlis RED*; Oxford Diffraction, 2006)	*k* = −12→11
*T*_min_ = 0.975, *T*_max_ = 0.982	*l* = −12→13
9430 measured reflections	

### Refinement

**Table:**

Refinement on *F*^2^	Secondary atom site location: difference Fourier map
Least-squares matrix: full	Hydrogen site location: inferred from neighbouring sites
*R*[*F*^2^ > 2σ(*F*^2^)] = 0.040	H atoms treated by a mixture of independent and constrained refinement
*wR*(*F*^2^) = 0.117	*w* = 1/[σ^2^(*F*_o_^2^) + (0.0674*P*)^2^ + 0.0667*P*] where *P* = (*F*_o_^2^ + 2*F*_c_^2^)/3
*S* = 1.09	(Δ/σ)_max_ < 0.001
3589 reflections	Δρ_max_ = 0.22 e Å^−^^3^
298 parameters	Δρ_min_ = −0.29 e Å^−^^3^
2 restraints	Extinction correction: *SHELXL97* (Sheldrick, 2008), Fc^*^=kFc[1+0.001xFc^2^λ^3^/sin(2θ)]^-1/4^
Primary atom site location: structure-invariant direct methods	Extinction coefficient: 0.008 (2)

### Special details

**Table:**

Geometry. All e.s.d.'s (except the e.s.d. in the dihedral angle between two l.s. planes) are estimated using the full covariance matrix. The cell e.s.d.'s are taken into account individually in the estimation of e.s.d.'s in distances, angles and torsion angles; correlations between e.s.d.'s in cell parameters are only used when they are defined by crystal symmetry. An approximate (isotropic) treatment of cell e.s.d.'s is used for estimating e.s.d.'s involving l.s. planes.
Refinement. Refinement of *F*^2^ against ALL reflections. The weighted *R*-factor *wR* and goodness of fit *S* are based on *F*^2^, conventional *R*-factors *R* are based on *F*, with *F* set to zero for negative *F*^2^. The threshold expression of *F*^2^ > σ(*F*^2^) is used only for calculating *R*-factors(gt) *etc*. and is not relevant to the choice of reflections for refinement. *R*-factors based on *F*^2^ are statistically about twice as large as those based on *F*, and *R*- factors based on ALL data will be even larger.

### Fractional atomic coordinates and isotropic or equivalent isotropic displacement parameters (Å^2^)

**Table:**

	*x*	*y*	*z*	*U*_iso_*/*U*_eq_	
O1	0.61774 (13)	−0.01358 (13)	0.71738 (12)	0.0408 (4)	
O2	1.11415 (15)	0.10175 (14)	0.58530 (13)	0.0488 (4)	
O3	0.76963 (14)	0.26997 (12)	0.97572 (11)	0.0373 (3)	
O4	0.43780 (16)	0.54591 (14)	0.67271 (14)	0.0557 (4)	
N1	0.32366 (15)	0.25812 (14)	0.94601 (14)	0.0331 (4)	
N2	0.71582 (15)	0.15648 (15)	0.76984 (13)	0.0293 (4)	
N3	0.84450 (15)	0.13134 (15)	0.70761 (13)	0.0306 (4)	
N4	1.00763 (16)	0.47023 (16)	1.23845 (14)	0.0368 (4)	
N5	0.70930 (14)	0.49236 (15)	0.96475 (13)	0.0268 (3)	
N6	0.62315 (15)	0.50278 (15)	0.86641 (13)	0.0283 (3)	
C1	0.2301 (2)	0.16377 (19)	0.97664 (17)	0.0355 (4)	
H1	0.1473	0.1808	1.0237	0.043*	
C2	0.24961 (19)	0.04281 (18)	0.94233 (17)	0.0329 (4)	
H2	0.1806	−0.0191	0.9640	0.039*	
C3	0.37305 (18)	0.01569 (17)	0.87553 (16)	0.0307 (4)	
H3	0.3890	−0.0656	0.8516	0.037*	
C4	0.47390 (18)	0.10983 (17)	0.84378 (15)	0.0267 (4)	
C5	0.44343 (18)	0.23048 (17)	0.88037 (16)	0.0300 (4)	
H5	0.5096	0.2951	0.8581	0.036*	
C6	0.60870 (18)	0.07795 (17)	0.77142 (16)	0.0288 (4)	
C7	0.94024 (19)	0.21070 (18)	0.71100 (16)	0.0316 (4)	
H7	0.9194	0.2776	0.7546	0.038*	
C8	1.07818 (19)	0.19826 (19)	0.64918 (16)	0.0337 (4)	
C9	1.18880 (17)	0.27331 (18)	0.64346 (15)	0.0294 (4)	
H9	1.1906	0.3450	0.6806	0.035*	
C10	1.3003 (2)	0.2241 (2)	0.57180 (17)	0.0404 (5)	
H10	1.3897	0.2571	0.5519	0.049*	
C11	1.2542 (2)	0.1210 (2)	0.53733 (18)	0.0458 (5)	
H11	1.3072	0.0695	0.4886	0.055*	
C12	1.0577 (2)	0.3466 (2)	1.30061 (17)	0.0386 (5)	
H12	1.1209	0.3400	1.3629	0.046*	
C13	1.0218 (2)	0.2281 (2)	1.27846 (18)	0.0397 (5)	
H13	1.0602	0.1442	1.3241	0.048*	
C14	0.92747 (19)	0.23689 (18)	1.18683 (16)	0.0340 (4)	
H14	0.9003	0.1585	1.1706	0.041*	
C15	0.87333 (17)	0.36337 (17)	1.11913 (15)	0.0262 (4)	
C16	0.91764 (18)	0.47580 (18)	1.14965 (16)	0.0318 (4)	
H16	0.8819	0.5611	1.1048	0.038*	
C17	0.77872 (18)	0.37120 (17)	1.01468 (15)	0.0267 (4)	
C18	0.57217 (18)	0.62304 (18)	0.81434 (16)	0.0293 (4)	
H18	0.5977	0.6954	0.8421	0.035*	
C19	0.47684 (18)	0.64921 (18)	0.71446 (16)	0.0316 (4)	
C20	0.41102 (18)	0.76644 (18)	0.65139 (15)	0.0302 (4)	
H20	0.4195	0.8518	0.6636	0.036*	
C21	0.32700 (19)	0.7377 (2)	0.56365 (17)	0.0369 (5)	
H21	0.2697	0.7994	0.5068	0.044*	
C22	0.3457 (2)	0.6057 (2)	0.5781 (2)	0.0546 (6)	
H22	0.3028	0.5586	0.5312	0.066*	
H2N	0.7125 (19)	0.2127 (17)	0.8202 (16)	0.035 (5)*	
H5N	0.713 (2)	0.5638 (17)	0.9912 (18)	0.043 (6)*	

### Atomic displacement parameters (Å^2^)

**Table:**

	*U*^11^	*U*^22^	*U*^33^	*U*^12^	*U*^13^	*U*^23^
O1	0.0418 (8)	0.0409 (8)	0.0502 (8)	−0.0061 (6)	0.0002 (6)	−0.0300 (7)
O2	0.0606 (9)	0.0428 (9)	0.0435 (8)	−0.0036 (7)	−0.0011 (7)	−0.0126 (7)
O3	0.0526 (8)	0.0267 (7)	0.0372 (7)	−0.0020 (6)	−0.0121 (6)	−0.0139 (6)
O4	0.0671 (10)	0.0386 (9)	0.0648 (10)	−0.0073 (7)	−0.0282 (8)	−0.0108 (7)
N1	0.0369 (9)	0.0286 (9)	0.0363 (9)	0.0003 (7)	−0.0034 (7)	−0.0137 (7)
N2	0.0332 (8)	0.0302 (9)	0.0293 (8)	−0.0044 (7)	−0.0002 (6)	−0.0158 (7)
N3	0.0342 (8)	0.0318 (9)	0.0273 (8)	−0.0021 (7)	−0.0019 (6)	−0.0105 (6)
N4	0.0373 (9)	0.0377 (10)	0.0394 (9)	−0.0021 (7)	−0.0085 (7)	−0.0152 (8)
N5	0.0296 (8)	0.0256 (9)	0.0286 (8)	−0.0016 (6)	−0.0047 (6)	−0.0126 (7)
N6	0.0290 (8)	0.0300 (9)	0.0282 (8)	−0.0037 (6)	−0.0042 (6)	−0.0105 (6)
C1	0.0370 (10)	0.0353 (11)	0.0354 (10)	−0.0026 (9)	0.0004 (8)	−0.0118 (9)
C2	0.0356 (10)	0.0267 (10)	0.0360 (10)	−0.0059 (8)	−0.0032 (8)	−0.0057 (8)
C3	0.0394 (10)	0.0224 (10)	0.0327 (10)	−0.0017 (8)	−0.0051 (8)	−0.0107 (8)
C4	0.0340 (10)	0.0250 (9)	0.0230 (9)	−0.0010 (7)	−0.0075 (7)	−0.0081 (7)
C5	0.0325 (10)	0.0282 (10)	0.0317 (10)	−0.0041 (8)	−0.0060 (8)	−0.0102 (8)
C6	0.0351 (10)	0.0265 (10)	0.0272 (9)	−0.0011 (8)	−0.0061 (7)	−0.0102 (8)
C7	0.0375 (10)	0.0317 (10)	0.0282 (10)	−0.0046 (8)	−0.0023 (8)	−0.0110 (8)
C8	0.0403 (11)	0.0367 (11)	0.0250 (9)	−0.0016 (8)	−0.0045 (8)	−0.0090 (8)
C9	0.0281 (9)	0.0377 (11)	0.0271 (9)	−0.0072 (8)	−0.0001 (7)	−0.0151 (8)
C10	0.0329 (10)	0.0528 (13)	0.0329 (10)	−0.0011 (9)	−0.0028 (8)	−0.0065 (9)
C11	0.0503 (13)	0.0470 (13)	0.0343 (11)	0.0125 (10)	0.0017 (9)	−0.0076 (10)
C12	0.0370 (11)	0.0459 (13)	0.0347 (11)	0.0010 (9)	−0.0094 (8)	−0.0132 (9)
C13	0.0501 (12)	0.0345 (11)	0.0344 (10)	0.0041 (9)	−0.0126 (9)	−0.0084 (8)
C14	0.0433 (11)	0.0295 (11)	0.0319 (10)	−0.0020 (8)	−0.0030 (8)	−0.0127 (8)
C15	0.0268 (9)	0.0270 (10)	0.0262 (9)	−0.0006 (7)	0.0017 (7)	−0.0105 (7)
C16	0.0348 (10)	0.0290 (10)	0.0329 (10)	−0.0011 (8)	−0.0060 (8)	−0.0098 (8)
C17	0.0309 (9)	0.0252 (10)	0.0256 (9)	−0.0040 (7)	0.0025 (7)	−0.0101 (7)
C18	0.0313 (9)	0.0289 (11)	0.0292 (10)	−0.0036 (8)	0.0001 (7)	−0.0103 (8)
C19	0.0312 (10)	0.0342 (11)	0.0302 (10)	−0.0073 (8)	0.0014 (7)	−0.0086 (8)
C20	0.0329 (10)	0.0320 (10)	0.0276 (9)	0.0022 (8)	−0.0036 (7)	−0.0125 (8)
C21	0.0351 (11)	0.0438 (13)	0.0311 (10)	−0.0036 (9)	−0.0056 (8)	−0.0067 (9)
C22	0.0612 (15)	0.0515 (15)	0.0581 (14)	−0.0118 (11)	−0.0262 (11)	−0.0167 (11)

### Geometric parameters (Å, °)

**Table:**

O1—C6	1.2258 (19)	C5—H5	0.9300
O2—C8	1.356 (2)	C7—C8	1.430 (2)
O2—C11	1.398 (2)	C7—H7	0.9300
O3—C17	1.2325 (19)	C8—C9	1.347 (2)
O4—C19	1.350 (2)	C9—C10	1.396 (2)
O4—C22	1.388 (3)	C9—H9	0.9300
N1—C1	1.337 (2)	C10—C11	1.333 (3)
N1—C5	1.338 (2)	C10—H10	0.9300
N2—C6	1.351 (2)	C11—H11	0.9300
N2—N3	1.381 (2)	C12—C13	1.377 (3)
N2—H2N	0.893 (14)	C12—H12	0.9300
N3—C7	1.285 (2)	C13—C14	1.379 (3)
N4—C12	1.333 (2)	C13—H13	0.9300
N4—C16	1.333 (2)	C14—C15	1.387 (2)
N5—C17	1.346 (2)	C14—H14	0.9300
N5—N6	1.383 (2)	C15—C16	1.390 (2)
N5—H5N	0.858 (15)	C15—C17	1.494 (2)
N6—C18	1.279 (2)	C16—H16	0.9300
C1—C2	1.376 (2)	C18—C19	1.432 (3)
C1—H1	0.9300	C18—H18	0.9300
C2—C3	1.371 (2)	C19—C20	1.340 (2)
C2—H2	0.9300	C20—C21	1.403 (2)
C3—C4	1.385 (2)	C20—H20	0.9300
C3—H3	0.9300	C21—C22	1.314 (3)
C4—C5	1.391 (2)	C21—H21	0.9300
C4—C6	1.499 (2)	C22—H22	0.9300
			
C8—O2—C11	105.59 (15)	C11—C10—C9	106.90 (17)
C19—O4—C22	105.42 (15)	C11—C10—H10	126.6
C1—N1—C5	117.26 (15)	C9—C10—H10	126.6
C6—N2—N3	119.18 (14)	C10—C11—O2	109.84 (16)
C6—N2—H2N	121.8 (12)	C10—C11—H11	125.1
N3—N2—H2N	117.9 (12)	O2—C11—H11	125.1
C7—N3—N2	114.99 (14)	N4—C12—C13	124.17 (18)
C12—N4—C16	116.25 (16)	N4—C12—H12	117.9
C17—N5—N6	118.33 (14)	C13—C12—H12	117.9
C17—N5—H5N	124.5 (14)	C12—C13—C14	118.32 (18)
N6—N5—H5N	117.2 (14)	C12—C13—H13	120.8
C18—N6—N5	115.34 (14)	C14—C13—H13	120.8
N1—C1—C2	123.62 (17)	C13—C14—C15	119.56 (17)
N1—C1—H1	118.2	C13—C14—H14	120.2
C2—C1—H1	118.2	C15—C14—H14	120.2
C3—C2—C1	118.45 (17)	C14—C15—C16	116.92 (16)
C3—C2—H2	120.8	C14—C15—C17	118.80 (15)
C1—C2—H2	120.8	C16—C15—C17	124.18 (16)
C2—C3—C4	119.73 (15)	N4—C16—C15	124.78 (17)
C2—C3—H3	120.1	N4—C16—H16	117.6
C4—C3—H3	120.1	C15—C16—H16	117.6
C3—C4—C5	117.67 (15)	O3—C17—N5	122.79 (16)
C3—C4—C6	118.95 (14)	O3—C17—C15	119.94 (15)
C5—C4—C6	123.37 (15)	N5—C17—C15	117.25 (14)
N1—C5—C4	123.25 (16)	N6—C18—C19	121.73 (16)
N1—C5—H5	118.4	N6—C18—H18	119.1
C4—C5—H5	118.4	C19—C18—H18	119.1
O1—C6—N2	123.49 (16)	C20—C19—O4	109.62 (16)
O1—C6—C4	120.77 (15)	C20—C19—C18	130.05 (17)
N2—C6—C4	115.74 (14)	O4—C19—C18	120.31 (16)
N3—C7—C8	121.36 (16)	C19—C20—C21	107.93 (17)
N3—C7—H7	119.3	C19—C20—H20	126.0
C8—C7—H7	119.3	C21—C20—H20	126.0
C9—C8—O2	109.95 (15)	C22—C21—C20	106.02 (18)
C9—C8—C7	128.47 (17)	C22—C21—H21	127.0
O2—C8—C7	121.59 (16)	C20—C21—H21	127.0
C8—C9—C10	107.72 (16)	C21—C22—O4	111.01 (18)
C8—C9—H9	126.1	C21—C22—H22	124.5
C10—C9—H9	126.1	O4—C22—H22	124.5
			
C6—N2—N3—C7	−179.93 (16)	C8—O2—C11—C10	0.4 (2)
C17—N5—N6—C18	−172.91 (14)	C16—N4—C12—C13	−0.2 (3)
C5—N1—C1—C2	−1.3 (3)	N4—C12—C13—C14	−0.4 (3)
N1—C1—C2—C3	1.6 (3)	C12—C13—C14—C15	0.8 (3)
C1—C2—C3—C4	−0.4 (3)	C13—C14—C15—C16	−0.7 (2)
C2—C3—C4—C5	−0.9 (2)	C13—C14—C15—C17	176.09 (15)
C2—C3—C4—C6	179.60 (16)	C12—N4—C16—C15	0.4 (3)
C1—N1—C5—C4	−0.1 (3)	C14—C15—C16—N4	0.1 (3)
C3—C4—C5—N1	1.2 (3)	C17—C15—C16—N4	−176.49 (15)
C6—C4—C5—N1	−179.32 (16)	N6—N5—C17—O3	0.7 (2)
N3—N2—C6—O1	−2.0 (3)	N6—N5—C17—C15	178.83 (13)
N3—N2—C6—C4	178.51 (14)	C14—C15—C17—O3	−12.5 (2)
C3—C4—C6—O1	15.9 (2)	C16—C15—C17—O3	163.98 (16)
C5—C4—C6—O1	−163.54 (17)	C14—C15—C17—N5	169.29 (15)
C3—C4—C6—N2	−164.56 (15)	C16—C15—C17—N5	−14.2 (2)
C5—C4—C6—N2	16.0 (2)	N5—N6—C18—C19	−177.59 (14)
N2—N3—C7—C8	−179.07 (15)	C22—O4—C19—C20	1.1 (2)
C11—O2—C8—C9	−0.6 (2)	C22—O4—C19—C18	179.52 (16)
C11—O2—C8—C7	179.48 (16)	N6—C18—C19—C20	178.28 (17)
N3—C7—C8—C9	179.63 (18)	N6—C18—C19—O4	0.2 (2)
N3—C7—C8—O2	−0.5 (3)	O4—C19—C20—C21	−0.8 (2)
O2—C8—C9—C10	0.6 (2)	C18—C19—C20—C21	−179.05 (17)
C7—C8—C9—C10	−179.48 (18)	C19—C20—C21—C22	0.2 (2)
C8—C9—C10—C11	−0.4 (2)	C20—C21—C22—O4	0.5 (2)
C9—C10—C11—O2	0.0 (2)	C19—O4—C22—C21	−1.0 (2)

### Hydrogen-bond geometry (Å, °)

**Table:**

Cg1 and Cg2 are the centroids of the O4/C19–C22 and O2/C8–C11 rings, respectively.

**Table:**

*D*—H···*A*	*D*—H	H···*A*	*D*···*A*	*D*—H···*A*
N5—H5N···N1^i^	0.86 (2)	2.10 (2)	2.944 (2)	169.(2)
N2—H2N···O3	0.89 (1)	2.08 (2)	2.9017 (19)	154.(2)
C21—H21···N3^ii^	0.93	2.58	3.410 (3)	149.
C16—H16···N1^i^	0.93	2.53	3.370 (3)	150.
C11—H11···O1^iii^	0.93	2.51	3.365 (2)	153.
C2—H2···O3^iv^	0.93	2.49	3.116 (2)	125.
C10—H10···Cg1^v^	0.93	2.78	3.594 (2)	146
C12—H12···Cg2^vi^	0.93	2.75	3.520 (2)	141

Symmetry codes: (i) −*x*+1, −*y*+1, −*z*+2; (ii) −*x*+1, −*y*+1, −*z*+1; (iii) −*x*+2, −*y*, −*z*+1; (iv) −*x*+1, −*y*, −*z*+2; (v) −*x*+2, −*y*+1, −*z*+1; (vi) *x*, *y*, *z*+1.
